# Senecavirus A as an Oncolytic Virus: Prospects, Challenges and Development Directions

**DOI:** 10.3389/fonc.2022.839536

**Published:** 2022-03-17

**Authors:** Dankun Luo, Haiwei Wang, Qiang Wang, Wenping Liang, Bo Liu, Dongbo Xue, Yang Yang, Biao Ma

**Affiliations:** ^1^ Department of General Surgery, The First Affiliated Hospital of Harbin Medical University, Harbin, China; ^2^ Key Laboratory of Hepatosplenic Surgery, Ministry of Education, The First Affiliated Hospital of Harbin Medical University, Harbin, China; ^3^ State Key Laboratory of Veterinary Biotechnology, Harbin Veterinary Research Institute, Chinese Academy of Agricultural Sciences, Harbin, China; ^4^ Departments of Biochemistry and Molecular Biology and Oncology, Arnie Charbonneau Cancer Institute, University of Calgary, Calgary, AB, Canada

**Keywords:** senecavirus A, oncolytic virus, cancer, virotherapy, immunotherapy, combination therapy

## Abstract

Oncolytic viruses have the capacity to selectively kill infected tumor cells and trigger protective immunity. As such, oncolytic virotherapy has become a promising immunotherapy strategy against cancer. A variety of viruses from different families have been proven to have oncolytic potential. Senecavirus A (SVA) was the first picornavirus to be tested in humans for its oncolytic potential and was shown to penetrate solid tumors through the vascular system. SVA displays several properties that make it a suitable model, such as its inability to integrate into human genome DNA and the absence of any viral-encoded oncogenes. In addition, genetic engineering of SVA based on the manipulation of infectious clones facilitates the development of recombinant viruses with improved therapeutic indexes to satisfy the criteria of safety and efficacy regulations. This review summarizes the current knowledge and strategies of genetic engineering for SVA, and addresses the current challenges and future directions of SVA as an oncolytic agent.

## Introduction

Compared with the development of traditional, chemotherapy-based treatment approaches against cancer, immunotherapy has proven to be a rapidly evolving field. In particular, cancer immunotherapy approaches often focus on the stimulation or enhancement of the host’s anti-tumor immune response to achieve a desired outcome. Among the different approaches in immunotherapeutics, oncolytic virus therapy (OVT), which relies on using viruses that selectively target transformed cells, has become a promising strategy for the treatment of cancer. Due to the natural defect in the type I interferon signaling pathway in most cancer cells, oncolytic viruses (OVs) can selectively infect and kill cancer cells lacking these canonical innate immune responses (1). Although OVs cannot infect non-cancerous cells due to their functional immune responses, these viruses can infect tumor cells and induce cytopathic effects that ultimately result in their death and the release of tumor-associated antigens. To date, the proposed mechanism for how OVs destroy tumor cells appears to be by directly inducing the lysis of the infected tumor cell, which subsequently triggers an immune response against the tumor to facilitate its removal.

## Genome Organization and Viral Replication of SVA

**Figure 1:**
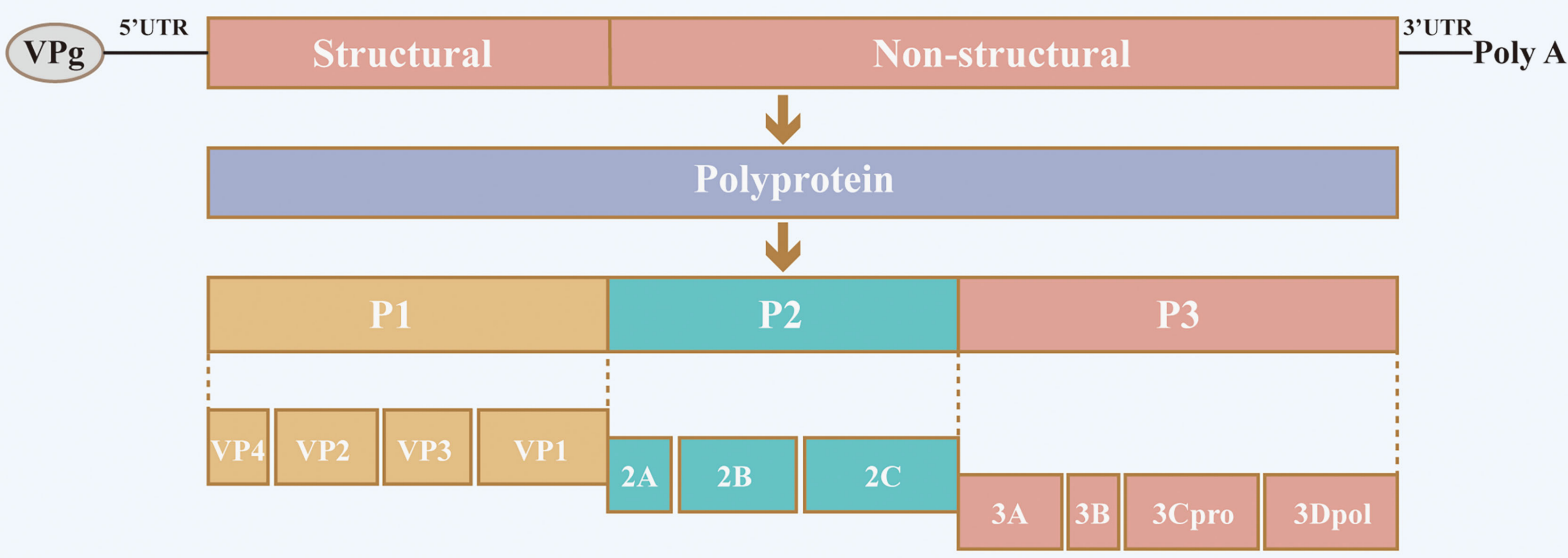
Schematic diagram of SVA structure and genome organization. The SVA genome comprises a large single open reading frame (ORF) flanked by highly structured 5′ and 3′ untranslated regions (UTR). The single ORF is translated into a single polyprotein, which is processed by viral proteases into multiple protein products P1 (VP4, VP2, VP3 and VP1), P2 (2A, 2B and 2C) and P3 (3A, 3B, 3Cpro and 3Dpol).

Senecavirus A (SVA), formerly known as Seneca Valley virus (SVV), belongs to the Seneca virus genus within the *Picornaviridae* family. The virus was first isolated incidentally in 2002 as a contaminant in a culture of PER.C6 transformed retinoblastoma cells (2, 3). Similar to other picornaviruses, the genome of SVA is a single-stranded, positive-stranded RNA composed of a large single open reading frame (ORF) flanked by highly structured 5′ and 3′ untranslated regions (UTR) that contain *cis*-acting elements essential for viral transcription, translation, and replication (**Figure 1**). The genome is translated into a single polyprotein which is processed by viral proteases into multiple protein products P1 (VP4, VP2, VP3 and VP1), P2 (2A, 2B and 2C) and P3 (3A, 3B, 3Cpro and 3Dpol) (2).

## SVA as a Natural Oncolytic Virus

The SVA genome is incapable of integrating into human genomic DNA during its infection life cycle, does not encode oncogenes, and is easy to modify through genetic engineering. These and other properties make this virus an attractive choice as a potential oncolytic picornavirus (4). Not surprisingly, the selective oncolytic activity of SVA is determined by its affinity towards specific cell receptors. Specifically, the infection of tumor cells by SVA depends on the presence of a receptor molecule highly expressed on their surface known as tumor endothelial marker 8 (TEM8) (5). As demonstrated by cell-based assays, SVA uses cellular TEM8 to gain entry into cells, and cryo-electron microscopy has further confirmed this conclusion by directly showing the structure of the SVA and TEM8 as a complex (6, 7). Additionally, SVA infection of pigs caused vesicular lesions are found on the snout, lips, or coronary bands. Infection of piglets with SVA can lead to weakness, lethargy or diarrhea (8). Pigs have been proven to serve as the primary hosts for SVA, although buffalo infection has been reported. SVA displays excellent safety conditions as a promising, highly-targetable oncolytic virus when compared to CBV3 and PV. SVA has shown anti-tumor efficacy in a variety of cancers, including medulloblastoma (9), retinoblastoma (10), glioma (11), and lung small cell carcinoma—highlighting its potential as a treatment against multiple malignancies (12). SVA as an oncolytic virus achieved satisfactory efficacy in a first-in-human phase I clinical trial in patients with small-cell lung cancer (SCLC) (13). Currently, the potential of SVA as an oncolytic virus is being tested in phase II clinical trials in patients with SCLC (12).However, patients with extensive-stage SCLC did not benefit from NTX-010 treatment after chemotherapy with a platinum doublet (12).

## Mechanisms of SVA Infection of Tumor Cells

### TEM8 Mediated Virus Replication and Reinfection of Neighboring Tumor Cells

The TEM8 receptor was first discovered as a tumor endothelial marker (14). This protein was also shown to serve as one of the receptors for the anthrax toxin, giving it its alternate name: anthrax toxin receptor 1 (ANTXR1) (15). It has been shown that N-glycosylation in ANTXR1 is a necessary post-translational modification for establishing stable interactions with SVA (16). Comparisons between a variety of tumor cells suggest increased expression levels of TEM8 compared to non-cancerous cells, and these high expression levels are often negatively correlated with patient prognosis (17–20). TEM8 is also widely expressed in the tumor-related stroma where it mediates tumor angiogenesis (21, 22), which provides oxygen, nutrition, and metastasis pathways for tumor cells (23). As such, TEM8 is an attractive potential target for antibody-based therapy. An anti-TEM8 antibody constructed by Chaudhary et al. (21) has been shown to slow tumor growth in mice and inhibit tumor-induced angiogenesis, but this monotherapy has not resulted in significant tumor regression (21, 24). A recent preclinical study showed that an antibody-drug conjugate (ADC) construct using monomethyl auristatin E (MMAE) coupled with an anti-TEM8 antibody could successfully induce an effective and dose-dependent anti-tumor response (4). This anti-tumor mechanism lies in the direct killing of TEM8+ tumor cells as well as intracellular proteases separating the MMAE and the P-glycoprotein drug transporter released into the tumor microenvironment when ADC is endocytosed by TEM8-expressing stromal cells, providing free MMAEs that can further kill tumor cells through the bystander effect (22, 25). In addition, human antibody-like molecules TEM8-Fc fusion protein, and TEM8 chimeric antigen receptor (CAR) T cell therapy have shown promising anti-tumor activity (26, 27). Nonetheless, these results are confounded by the cross-reactivity of the TEM8 therapeutic antibody with its paralog, ANTXR2, and thus the inability to uncouple observed phenotypic responses against the therapeutic agents (5).

It is important to note that recent studies have found that SVA can specifically target tumor cells that express ANTXR1 without causing any off-target effects on normal cells with highly expressed ANTXR2. Moreover, most of the receptor residues that contribute to virus-receptor interactions are not conserved between the two receptors (6). This specificity ensures that TEM8, not ANTXR2, can selectively act as the direct binding entry receptor for SVA (5, 7). Not surprisingly, the high expression levels of TEM8 seen in cancerous cells correlate with the susceptibility of particular tumor cells to SVA infection, and the introduction of exogenous TEM8 into otherwise SVA-insensitive tumor cells can significantly increase viral entry and the proportion of cells killed-paving a new way for optimizing TEM8-targeted therapies using oncolytic viruses (5).

## Regulation of Innate Immune Response

Compared with other tumor treatments, one of the therapeutic advantages of OV is that it can trigger the host’s anti-tumor immune response (28). For example, when using PVSRIPO to kill tumor cells, the lysed cells can release tumor-associated antigens (TAAs), pathogen‐associated molecular patterns PAMPs) and damage‐associated molecular patterns (DAMPs), and promote the activity of antigen-presenting cells (APCs). The cellular response to the virus induces a sustained type I interferon (IFN) response, co-stimulatory molecule expression, and cytokine production in the nearby environment. Ultimately, this inflammation and antigenic stimuli will positively affect the production of anti-tumor T cell populations that can directly lyse cancer cells (29). Such observations have been confirmed, for example, when using an attenuated live measles virus (MV) strain to treat malignant pleural mesothelioma (30).

Upon infection, cellular antiviral responses against the OV can stimulate the IFN pathway and up-regulate a variety of chemokines, resulting in the recruitment of additional T cells infiltrates that further amplify the immunosuppressive microenvironment and induce PD-L1 expression (31). As a result, the combination of OV with immune checkpoint inhibitors (ICIs) can significantly increase the efficacy of OV treatment (32). Unfortunately, the role of SVA-based viral therapy in activating anti-tumor immunity has received much less attention compared to its intrinsic oncolytic potential.

The innate immune response is the first line of defense against pathogen invasion. The body’s strong antiviral response is the main reason oncolytic viruses the efficacy of OV has not been as good as initially thought (33). Upon recognizing viral signatures by diverse host pattern recognition receptors (PRR), cellular antiviral programming commences with the induction of type I IFNs that signal to neighbor cells to stimulate the expression of IFN-stimulated genes (ISG) that can exert a variety of direct or indirect antiviral functions (34, 35). To cope with such antiviral mechanisms, most viruses have co-evolved to escape or subvert these responses. Although SVA is naturally sensitive to the antiviral effects of type I IFNs (36), the virus can still evade the immune response by interfering with the induction and signaling of the type I IFN pathway in multiple ways. For example, SVA 3Cpro uses its protease activity to directly degrade the key transcription factors IRF3 and IRF7 or target adapter proteins MAVS, TRIF, and TANK in the type I IFN pathway, dampening IFN synthesis (37). In addition, SVA can also promote its survival by inhibiting the host ubiquitination system that regulates many aspects of the cell, including immune signaling (38, 39). For example, SVA 3Cpro uses its deubiquitinase activity to deubiquitinate the key type I IFN signaling molecules RIG-I, TBK1, and TRAF3, evading antiviral immunity and promoting self-replication as a result (38).

During OV treatment, the host IFN pathway behaves like a double-edged sword. On the one hand, it can activate the host’s anti-tumor immunity and maintain a longer-term tumor treatment effect, while on the other, it can cause OV to be prematurely cleared and lose the curative effect in the host. Deepening our understanding of the spatial and temporal interactions between the OV and the body’s immune system can provide new ideas for the improvement of OV variants that are less immunogenic yet retain their ability to selectively kill tumor cells.

## SVA Infection-Induced Cell Death, Including Apoptosis, Autophagy, or Pyroptosis

Death is the final outcome of a cell, but how a cell reaches this state is a complicated process. Current research has identified a variety of cell death pathways, which are mainly divided into programmed cell death, or apoptosis, and non-programmed necrosis (40). Different cell death pathways have specific regulatory factors and effectors molecules that contribute to the diverse ways by which a cell can ultimately die. As such, these regulators represent potential therapeutic targets that are expected to improve the prognosis of patients with certain diseases.

It has been demonstrated that viruses such as SVA can induce apoptosis as means to achieve effective virus transmission (41–43). Significant levels of apoptosis have been observed during SVA infection both *in vivo* and *in vitro* (44, 45), often seen in the late stages of infection. This may be related to inhibition of the transcription factor NF-κB, one of the main players in regulating host cell apoptosis and pro-inflammatory response (46, 47), in both the middle and late stages of infection (44). One of the key subunits of NF-κB, p65, can inhibit both virus replication and apoptosis in cells by inducing the expression of key proteins involved in regulating immune responses and apoptosis. In the later stages of SVA infection, the viral 3Cpro activates caspase to cleave p65, resulting in a significant decrease in the transcriptional activity of NF-κB (44).Moreover, apoptotic cells can promote the release and spread of SVA from infected cells, thereby facilitating a broader “tumor-killing” effect within the tumor (44). Apoptosis has two different apoptotic pathways with the same trend (48). The extrinsic pathway is triggered by extracellular stimuli and regulated by membrane death receptors, while the intrinsic pathway is triggered by cellular stress and regulated by mitochondrial-related proteins that cause the release of cytochrome C into the cytoplasm. Both pathways eventually lead to the activation of caspase-3, which subsequently cleaves various substrates that ultimately lead to nuclear fragmentation prior to apoptosis (48). SVA infection of 293T cells activated caspase-9, caspase-8, and caspase-3 in a time-dependent manner. Caspase-9 and caspase-8 represent the activation of intrinsic and extrinsic pathways, respectively, indicating that SVA can initiate apoptosis by both pathways. This process is mainly mediated by the 2C and 3Cpro proteins of SVA (45). The SVA 2C protein interacts with the C-terminal region of the anti-apoptotic protein Bcl-xL, thereby interfering with the canonical interaction between Bcl-xL and Bax, increasing the levels of endogenous Bax in the mitochondria, and inducing intrinsic cell apoptosis. On the other hand, the 3C protein seems to use its protease activity to induce apoptosis through both intrinsic mitochondrial pathway and extrinsic death receptors signaling pathways (45). However, the specific mechanism for 3Cpro regulation of apoptosis needs to be further explored.

Interestingly, the type of cell death induced by SVA appears to be species selective (49). In human cell lines (e.g., H1299, 293T), SVA infection mainly mediates apoptosis, while in porcine cells (e.g., SK6), SVA infection induces caspase-dependent and independent pyroptosis. This may be related to differences in gasdermin D (GSDMD), a key executor molecule of pyroptosis with pore-forming ability, between different species (50). A glutamine residue in porcine GSDMD, Q277, has a high affinity for SVA 3Cpro, which can cleave GSDMD directly leading to the exposure of its N-terminal domain with pore-forming activity (49). In addition, SVA 3Cpro cleaves the poly(A) binding protein cytoplasmic 1 (PABPC1), a protein that can inhibit viral replication in host cells, to promote SVA replication (51). After being assembled into plasma membrane pores, it can release biologically active substances and other cellular contents to kill cells (40, 52). Accordingly, genetic engineering of SVA with 3C protein modifications to induce tumor cells pyroptosis could be a strategy in improving SVA oncolytic activity in human cells.

Autophagy is usually an adaptive response that seeks homeostasis by isolating and degrading damaged and harmful cellular and foreign components in double-membrane vesicles. However, autophagy is also involved in cell death under certain circumstances (40, 48). Several studies have found that SVA infection can activate autophagy to complete its replication cycle rather than as a means to kill the infected cell (9, 53). SVA infection induces autophagy, at least in part, by activating the PKR-like ER protein kinase (PERK) and the activating transcription factor 6 (ATF6) pathways of the unfolded protein response (UPR) (53). The autophagy mechanism induced by SVA is different from the autophagy activated by traditional rapamycin. Activation of autophagy by rapamycin will also disrupt the autophagy process induced by SVA (9, 54). Interestingly, this phenomenon seems to be species-specific (55). Autophagy inducers can inhibit the process of virus-induced autophagy in multiple human cell lines, resulting in reduced SVA replication levels. However, SVA can promote the proliferation and spread of the virus by inducing autophagy in porcine cells. The molecular mechanisms of these species-specific differences need to be further characterized (9, 53, 55).

**Figure 2:**
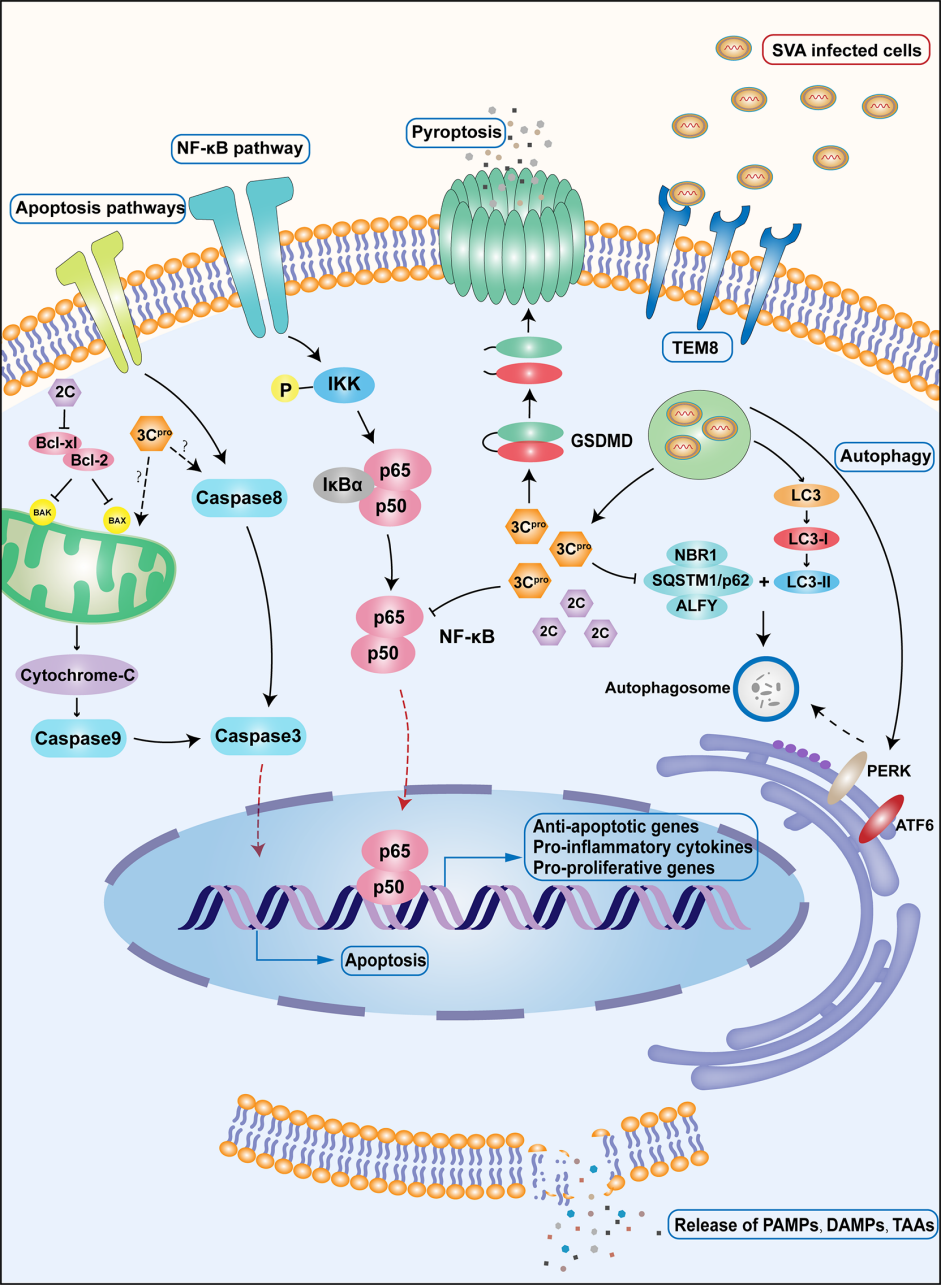
Important immune and apoptotic pathways during SVA infection. Upon SVA delivery into the body by either intravenous or local injection, SVA first enters the cell by recognizing the specific receptor TEM8.SVA uncoating releases the viral genome into the cell, which is immediately translated and processed into the corresponding viral proteins. Among the proteins produced, 3Cpro promotes the cleavage of the important subunit p65 of NF-κB, leading to a decrease in NF-κB transcriptional activity and, as a consequence, induction of apoptosis. A different protein, 2C, interacts with the C-terminal region of Bcl-xL, which interferes with the interaction between Bcl-xL and Bax, increases the level of Bax in the mitochondria, and induces apoptosis. 3Cpro can also induce cell apoptosis through intrinsic and extrinsic death pathways. The specific mechanism remains to be studied. In porcine cells, 3Cpro can also directly lyse GSDMD and induce pyroptosis. SVA can induce autophagy through PERK pathway and ATF6 pathway after infecting cells. The 3Cpro protein can inhibit selective autophagy by cleaving the autophagy receptor SQSTM1/p62. Cells that die after being infected by SVA will release large amounts of TAAs, DAMP, and PAMP, stimulating a wider range of cellular responses.

Viruses have evolved various strategies to counteract autophagy-related antiviral responses in the host. SQSTM1/p62 is an autophagy receptor that can present ubiquitinated substrates to autophagosomes to complete phagocytosis and an important component of the cellular antiviral mechanism (56, 57). In SVA-infected cells, overexpressed SQSTM1 promotes virus degradation by targeting SVA’s capsid proteins VP1 and VP3, ultimately inhibiting the spread of SVA (55). However, current studies have found that the 3Cpro protease of SVA can inhibit SQSTM1-mediated selective autophagy by cleaving SQSTM1, eliminating its antiviral effect (55). In addition, the 3A protein of SVA also has the function of inhibiting the host’s antiviral function. Additionally, studies have found that 3A proteins from multiple picornaviruses, including that of SVA, can degrade G3BP1, a multifunctional protein that participates in a variety of host antiviral responses and inhibit the PRR signaling pathway mediated by it (58) (**Figure 2**).

SVA induces cell death through a variety of pathways, but most of these mechanisms are based on those observed in non-tumor cells. Although SVAs have achieved satisfactory efficacy as OVs, whether their mechanisms of killing tumor cells are consistent with the mechanisms of infecting non-tumor cells requires further research. Understanding the tumor cell death caused by SVA will help rational design OV with more efficacy to improve the prognosis of cancer patients.

## Engineering Oncolytic Viruses to Enhance Virus Safety and Oncolytic Capacity Directed Evolution: Through the Passage of Virus in Specific Tumor Cells, the Virus Can Selectively Target Tumor Cells

**Figure 3:**
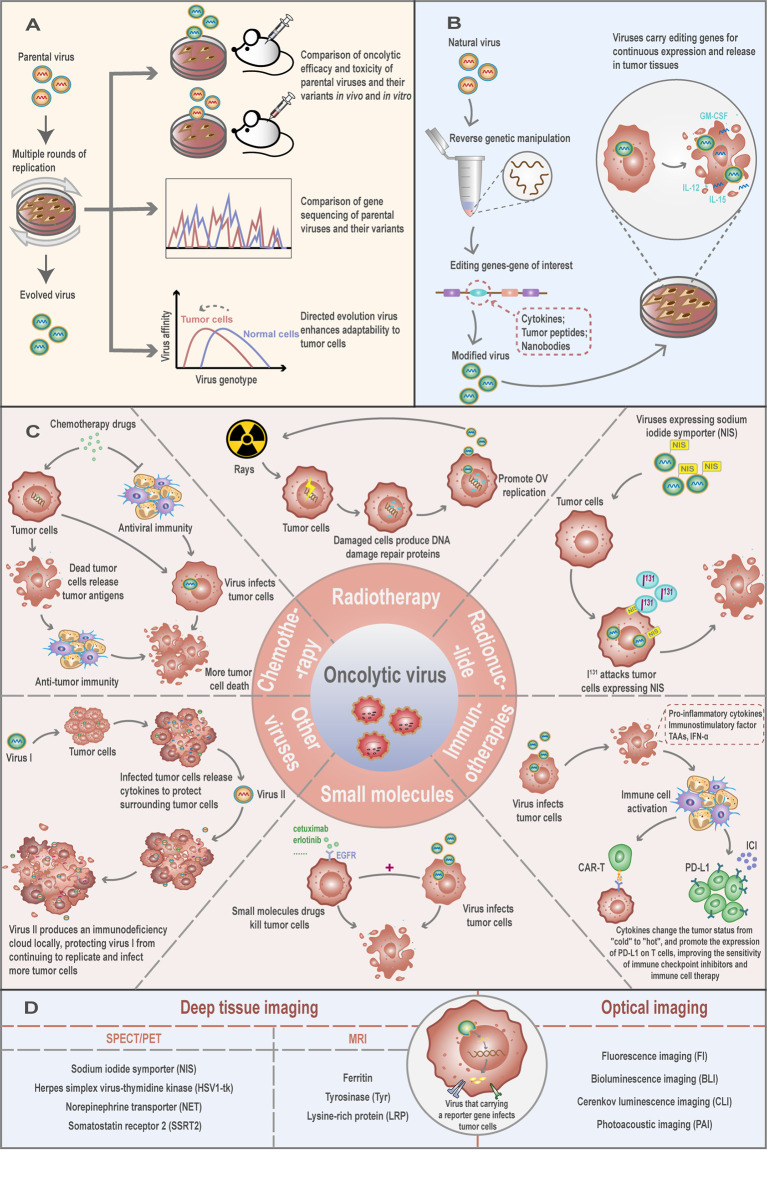
Engineering oncolytic viruses to enhance virus safety and oncolytic capacity. **(A)** Directed evolution: through the passage of virus in specific tumor cells, a comparison of oncolytic efficacy and toxicity of parental viruses and their variants *in vivo* and *in vitro*, or comparison of gene sequencing or screening viruses that are adaptable to tumor cells, can improve the selectivity of the viruses to tumor cells. **(B)** Oncolytic virus is a powerful vector that loads the interest gene like cytokines, tumor peptides, or nanobodies into the viral genome by genetic engineering. In this system, the OV carries these foreign genes into the host cell for expression and exert their biological functions. **(C)** The necessity and strategic combinations of an oncolytic virus with chemotherapy and other biological strategies. **(D)** Oncolytic virus encoding reporter genes for *in vivo* molecular imaging.

Under the effects of natural selection, viruses continue to evolve and adapt to changes in the environment. The RNA-dependent RNA polymerases of RNA viruses lack a proof-reading mechanism, leading to progeny viruses displaying an unusually high mutation rate. As a consequence, RNA viruses often exist within their host in the form of a population referred to as “quasi-species” (59, 60). Viruses within the population are genomically and phenotypically different, implying that OV may have different tumor-killing effects. Based on this feature, some studies proposed using “directed evolution” through the serial passage of OV on human solid tumor cell lines to screen out the most effective OV in improving the therapeutic effect (61, 62) (**Figure 3A**). This method uses the complexity and diversity of human tumor cells to direct the selective and efficient evolution of viruses and is especially suitable for RNA viruses with a high spontaneous mutation rate (63). For instance, it has been shown that, after the continuous passage of recombinantly replicated VSV (rrVSV) in D2F2/E2 cells, a virus subtype with higher infection efficiency and stability to the cell can be produced (64). This suggests that specific virus subspecies can be constructed for specific cell types through the “directed evolution” to improve the adaptability and tumor selectivity of OV.

Directed evolution is also expected to improve the spread of viruses in tumors. For example, it is known that the Newcastle disease virus (NDV) is highly sensitive to the human fibrosarcoma HT1080 cell line, but the limitation of the intratumoral barrier results in the inability of the virus to spread sufficiently and for the tumor to lyse completely. Therefore, two rounds of screening for NDV in tumor xenografts of HT1080 have resulted in the isolation of a virus with higher proliferation potential than the parent (65). In comparison with viruses modified by genetic engineering, viruses obtained by “directed evolution” should retain their underlying molecular killing mechanisms, which overcomes some limitations caused by the complexity of virus-host interactions and provides new ideas for virus improvement (63). Therefore, directed evolution could be used to improve the adaptability and selectivity of SVA in certain tumors.

## Oncolytic Virus as a Vector Expressing Cytokines, Tumor Peptides, or Nanobodies as Anti-Tumor Therapeutics

Viruses can be powerful vectors with the capacity of carrying genes of interest in their viral genome through genetic engineering. Foreign genes can be carried by the OV into host cells for their expression (**Figure 3B**). Some studies have shown that combining viral therapy with cytokines and using the host’s immune function to assist the virus in the rejection and destruction of tumors can significantly improve the anti-tumor effect of OV. The first cytokine used in such combination, GM-CSF, has achieved a curative effect that exceeded the estimate. Kim et al. (66) constructed a vaccinia virus that can express human GM-CSF that displays strong tumor-killing activity in liver primary tumor models and lung metastasis models. We have constructed a recombinant SVA expressing GM-CSF, which is under evaluation *in vitro* and *in vivo* (unpublished data). This powerful systemic anti-tumor response is the consequence of the two components of the vector: the OV and the exogenous cytokine. This type of modified virus with a variety of action mechanisms expands the use of OV in a new direction, and additional cytokines are currently being applied to the combination therapy of viruses. For example, the vaccinia virus expressing the *IL-24* gene has been found to inhibit tumor cell growth by inducing oncolysis and apoptosis and stimulating anti-tumor immunity in breast cancer and colorectal cancer (67, 68). In addition, such modified viruses are expected to improve the “immune desert” microenvironment of tumors. For instance, it was found that the interaction of IL-23, a cytokine that can prolong viral persistence, and oncolytic vaccinia virus further increased the expression of Th1 chemokines and anti-tumor factors (69). Thus, increased infiltration of activated T cells and the ratio of CD8+-to-Treg cells further transform the immunosuppressive tumor microenvironment, exerting a powerful anti-tumor effect.

OV can also enhance the synergistic anti-tumor effect by loading multiple cytokines. The cytokine GM-CSF can function in the recruitment of DCs and NK cells as well as in the induction of tumor-specific cytotoxic T lymphocytes, which are important in inducing specific and durable anti-tumor immunity. In turn, IL-24 displays anti-tumor activity and can inhibit angiogenesis. Oncolytic viruses can be used as vectors to target these anti-tumor genes to the tumor area, forming a synergistic anti-tumor effect. Given these scenarios, a recombinant vaccinia virus co-expressing GM-CSF and IL-24 has shown stronger anti-tumor activity than a virus carrying either GM-CSF or IL-24 alone (70). Specifically, the effects of the vaccinia virus encoding both IL-7 and IL-12 are related to its ability to improve the inflammatory immune status of the tumor microenvironment and the sensitivity to systemic anti-PD-1 and ctla4. Such a setting provides a strategy to overcome tumor resistance to immunotherapy (71).

Although the virus can mediate tumor cell lysis and release TAAs to stimulate the activation of anti-tumor-specific immune response in the host, it is often not enough to induce sufficient tumor-specific CD8+ T cell immune response. Studies have found that when one or more TAAs are artificially encoded into the OV genome, it is expected to enhance the response level of immune cells such as T cells, overcome immune escape, and mediate tumor-specific immune responses to participate in anti-tumor work (72). In a study by Vries et al (73), the growth of mouse breast tumors driven by HER2/neu resulted in the infiltration of intratumoral and systemic myeloid-derived suppressor cells (MDSCs), leading to immune escape and lack of systemic immunity. When treated with a recombinant vaccinia virus encoding GM-CSF, the infection does not induce systemic immunity nor cause tumor regression. However, when combined with the vaccinia virus encoding HER2/neu and administered to the tumor microenvironment, mice develop systemic anti-neu immunity, display significantly reduced tumor volume and systemic MDSCs infiltration, and exhibit a significant anti-tumor response. In a different study, the MG1-Maraba virus expressing prostate six transmembrane antigen (STEAP) exerted oncolytic activity on mouse prostate tumors, and at the same time produced specific CD8+ T cell responses against multiple STEAP epitopes (74). The production of these CD8+ T cells breaks the immune tolerance to the antigen, transforms the immunosuppressive microenvironment and prolongs the survival rate of mice with advanced prostate cancer models (74). These results show that increasing the number of TAAs in the tumor microenvironment is expected to transform the “cold” immune status into “hot,” which can be clinically verified in the future.

At present, OV and antibody treatments are two emerging fields in cancer immunotherapy. Both of them can be obtained through molecular engineering, which significantly improves tumor targeting and killing efficacy (75). The two therapies are combined to deliver antibodies from the amplified viral genome to the tumor by infecting host cells (75). It was found that mice infected with oncolytic vaccinia virus expressing anti-EGFR single-chain antibody (GLV-1h442) show significantly slower tumor growth *in vivo* than mice infected with empty oncolytic vaccinia virus (GLV- 1h68) alone (76). Furthermore, the oncolytic virus vector can be used to complete the co-expression of antibodies with multiple functions in tumors, including direct-killing tumor cell antibodies, anti-angiogenic antibodies, tumor stromal cell-killing antibodies, or antibodies that can activate immune responses, to achieve a more powerful tumor growth inhibitory power (75). SVA possess a positive-strand genomic RNA of ~7.5kb, so it can tolerate gene insertion of less than 800bp in length, so SVA could express single-chain antibody instead of regular monoclonal antibodies.

## The Necessity and Strategic Combinations of an Oncolytic Virus With Chemotherapy and Other Oncolytic Viruses

Studies have shown that a reasonable combination of chemical and biological strategies can produce synergistic effects with OV therapy (**Figure 3C**). At present, a variety of combined treatment strategies have achieved curative effects. For example, the synergistic application of temozolomide, an alkylating agent, commonly used in chemotherapy, with an oncolytic adenovirus enhanced the death of xenograft tumor cells of H441 lung cancer and A375 melanoma (77, 78). This synergistic effect is mediated at least in part by promoting virus replication, accelerating cell apoptosis, and inducing autophagy and anti-tumor immune responses (79, 80). In addition, the alkylating agent cyclophosphamide has an immunosuppressive effect, which can reduce the rate of the virus being neutralized and prolong the action time of the virus (81). Cancer cells destroyed by cytotoxic chemotherapeutic drugs also release DAMPs and soluble antigens to promote the body’s anti-tumor immune response (82).

Combining radiotherapy with OV can result in increased tumor cell toxicity and enhanced virus replication ability in tumor cells. Radiation can promote cell cycle arrest and DNA repair in response to radiation damage. For example, a mutant herpes simplex virus (HSV) lacking the growth gene ICP34.5 can restore its replication function under the action of radiotherapy. This is due to the increased expression of Growth Arrest and DNA damage Protein 34 (GADD34), a DNA repair protein with significant homology with herpesviral ICP34.5, in cells under the action of radiation (83). In lung cancer cell lines, a 16-fold increase in HSV-1 titer was observed within seven days of infected cells after 2Gy radiation compared to infection alone. Notably, synergistic anti-tumor effects were also observed in “*in vivo*” experiments (83). This phenomenon has also been confirmed in cholangiocarcinoma (84).

In addition to promoting virus proliferation, the synergistic effect of radiotherapy and OV can also increase the apoptotic response of cells. A study found that in irradiated cells infected with NV1023, an engineered HSV-1, the percentage of apoptosis was significantly higher than that of irradiated cells without virus treatment (85). Similarly, OV has a synergistic effect with radionuclide therapy. For example, in multiple myeloma cells that are sensitive to radiation, the use of recombinant OV expressing the human sodium iodide symporter (NIS) gene in combination with the radioisotope iodine-131 (^131^I) can enhance the therapeutic effect. At the same time, the infected tumor cells can be imaged through planar scintillation scanning to understand the distribution and metastasis of tumor cells. This image-guided radio viral therapy is expected to become a promising new method for the treatment of radiation-sensitive tumors (86, 87).

Biologically targeted therapy is an emerging field of cancer therapy. Studies have found that a variety of biological therapies can obtain greater benefits when used in combination with oncolytic HSV. Cetuximab (anti-EGFR monoclonal antibody) and bevacizumab (anti-VEGFA monoclonal antibody) enhanced the distribution of the virus in the entire tumor by inhibiting angiogenesis, expanded the number of apoptotic cells, and successfully induced a synergistic anti-tumor effect (88, 89). Valosin protein inhibitor (VCP) cooperates with the M1 virus, a naturally occurring alphavirus that shows potent oncolytic activities against many cancers (90), to promote endoplasmic reticulum stress-induced apoptosis by inhibiting the IRE1a-XBP1 pathway. In particular, it has been shown to synergistically kill liver cancer cells in *in vitro* and *in vivo* experiments (91). In addition, OV can also enhance the effect of immunotherapy. ICIs often respond poorly in some tumors with a special “cold” immune microenvironment because the microenvironment itself lacks immune effector cells. Therefore, transforming the “cold” tumor microenvironment into a “hot” microenvironment is expected to improve the effectiveness of ICI. OV can target and kill cancer cells, release a large amount of TAAs and express pro-inflammatory and immune-stimulating cytokines, and promote the infiltration of anti-tumor immune cells such as activated T cells and NK cells in the local tumor microenvironment (92). Notably, OV infection can stimulate the secretion of type I IFN, leading to the up-regulation of PD-L1 in tumor cells and tumor-infiltrating immune cells, which significantly enhances the anti-tumor immune effect (93). The efficacy of Chimeric Antigen Receptor T Cell (CAR-T), a fusion molecule that combines specific antibodies and immune cell effector functions, has been affirmed in some hematological malignancies. However, due to the obstacles of the immunosuppressive microenvironment surrounding solid tumors, its efficacy in solid tumors is not satisfactory. But now there seems to be a better solution. The immunosuppressive microenvironment transformed by OV can promote the migration and survival of CAR-T cells in the tumor microenvironment, and overcome the challenges faced by its application in solid tumors (94). Additionally, the use of different mechanisms between viruses to kill cells and the combined application of different viruses can also play a synergistic anti-tumor effect. Boeuf et al. (95) hypothesize that when a virus infects tumor cells, the tumor cells secrete anti-virus-related factors to resist the virus’s invasion. Under this view, if a second virus is added at this time, the incoming virus can locally produce immunosuppressive factors to protect the first virus while it continues to search for permissive tumor cells to complete its replication. Therefore, understanding the optimal combinations of biological therapies for diverse cancers has great potential for the improvement and development of novel immunotherapies.

## Oncolytic Virus Encoding Reporter Genes for *In Vivo* Molecular Imaging

Reporter gene imaging (RGI) has the ability to noninvasively and continuously identify the target site of the virus and, by measuring the level of virus infection, it can provide information on the safety, effectiveness, and toxicity of the virus. This real-time tracking can also provide information on the dose and time of administration of the virus to optimize treatment, as well as the ability to detect tumor origin and metastasis. These provide new ways for the diagnosis and treatment of tumors in the future (96). At present, viral molecular imaging technologies are mainly divided into two categories: optical imaging and deep tissue imaging (96, 97) (**Figure 3D**). The principle relies on the integration of a reporter gene into the OV genome, allowing it to be monitored in the host upon translation. Ultimately, an external device is used to track these proteins to complete the imaging (97).

Optical imaging methods mainly include Fluorescence imaging (FI), Bioluminescence imaging (BLI), Cerenkov luminescence imaging (CLI), and Photoacoustic imaging (PAI) (97). At present, in the research of SVA, the use of optical imaging occupies the dominant position. For example, Poirier et al. (98) used a modified SVA that expresses the green fluorescent protein (GFP) reporter gene to determine the SCLC cell subpopulation that the virus is most selective towards. Liu et al. (99) replaced GFP with NanoLuc^®^ luciferase (NLuc) to construct a recombinant SVA that can effectively express this small luciferase enzyme. The authors found that this luciferase offers many advantages over fluorescent proteins and shows higher virus replication ability in SCLC cell lines. Unfortunately, the foreign genes in these viral genomes became unstable after several passages. In order to circumvent this problem, Wang et al (100) inserted the green fluorescent protein (iLOV), red fluorescent protein (RFP) or nanoluciferase (Nluc) gene at the junction between SVA 2A and 2B and, in addition, a stop-restart translation element T2A was inserted into the side of the 2B product of SVA to retain the natural coding sequence of the viral protein surrounding the foreign gene. The author found that SVA with the iLOV tag can maintain a high level of stable passage in cells. Previously, we developed a new reporter SVA that expresses enhanced green fluorescent protein (eGFP) and found that the viral RNA-dependent RNA polymerase (RdRp) plays a key role in eGFP retention (101). The reporter viruses could be used to for real-time visualization by bioluminescent tumor cells.

Since optical imaging relies on the light in the infrared, visible, or ultraviolet spectrum, deep tissue imaging cannot be performed. At present, various types of deep tissue reporter genes have been developed for OVT. Common reporter genes that rely on radionuclide imaging (SPECT/PET) mainly include encoding symporters/symporters like Sodium iodide symporter (NIS) and Norepinephrine transporter (NET), encoding enzymes like Herpes simplex virus thymidine kinase (HSV1-tk), and encoding the receptor-like Somatostatin receptor 2 (SSRT2) (96, 97). The other is a reporter gene that relies on functional/molecular magnetic resonance imaging (f/mMRI). Since f/mMRI is less sensitive than SPECT/PET, it is necessary to build a higher contrast between the target and the background to reduce this limitation (96). At present, the most effective reporter genes include encoding Ferritin, Tyrosinase (Tyr), and Lysine-rich protein (LRP). In the future, more new reporter genes need to be developed to promote the broader use of this imaging technology (97). Although an unprecedented breakthrough has been made in the field of RGI for OV, the direction of its breakthrough is still focused on the in-depth optimization of imaging sensitivity. RGI is an advanced technology that integrates diagnosis and treatment; hence, deepening our understanding of the therapeutic benefits of RGI will be key in maximizing the advantages of this technology.

## Current Challenges and Future Research Directions

The ideal OV should display non-pathogenic and optimal tumor-killing effects on the host. SVA has many unique advantages as an OV, even within the *Picornaviridae* family. SVA is small in genomic size, is non-enveloped, and has a short replication cycle-which aids in the quick distribution within the entire tumor. And as a single-stranded RNA virus, it lacks the intermediate DNA step when replicating in the host cell, avoiding any potential integration of the viral gene into the host gene and other frequent concerns when using viruses for biomedical purposes (33). As previously mentioned, SVA is relatively easy to produce in large quantities to supply a large number of clinical needs. In addition, SVA also has a special advantage: it can be administered intravenously without reducing its tumor-killing efficiency, which brings hope of a cure for tumors that have undergone distant metastasis (102). Reddy et al. injected SVA intravenously into nude mice with pre-established small cell lung cancer or retinoblastoma and found that both mice produced a complete and lasting tumor-killing response (33). Since SVA is not pathogenic to humans, there are virtually no neutralizing antibodies in human serum. Besides, SVA is not restricted by any components of human blood and does not produce toxicity to normal cells, which greatly improves the safety of intravenous medication (33). Besides, the intercellular transmission of SVA was mediated by exosomes (103). Importantly, the spleen is a secondary lymphoid organ that supports the effective activation of T cell responses and plays an important role in the production of tumor-killing T cells. SVA, through intravenous administration, can enter the spleen and stimulate the spleen immune response to establish a long-term anti-tumor immune environment for the body (28).

Although SVA has an encouraging effect on a variety of endocrine tumors clinically, there are still some challenges to developing an OV. SVA outbreak incidents have been reported in the United States (104), Brazil (105), China (106), Colombia (107), Thailand (108), and other countries in a decade, showing that SVA infection causes vesicular diseases and could be a potential threat to the global pig industry. Considering its high pathogenicity in pigs, we should first pay attention to the safety of SVA as an oncolytic virus. Sequencing analysis and phylogenetic tree results revealed that Senecavirus A strains share high amino acid (above 95%) similarities. Therefore, vaccines made from one field isolate could protect against all circulating strains. We suggest that the live attenuated vaccine candidate be used to develop a promising oncolytic virus. Upon repeated administration, the body will eventually produce neutralizing antibodies against SVA. In a phase I clinical trial, all 30 patients who received treatment developed neutralizing antibodies against the virus (13). Although the antibodies produced after the first dose of OV could hinder the efficacy of systemic administration in the treatment of metastatic tumors, it would not interfere with the local efficacy of intratumoral delivery (33, 109). The use of immunosuppressive agents before or during OV treatment can minimize the development of immune responses and delay the production of neutralizing antibodies and virus clearance (110). Shielding OV with synthetic polymers or exosomes can also reduce contact with the body’s immune surveillance cells during OV transportation (111).

Immune efficacy can also be increased by enhancing the killing efficiency of the virus before the body produces neutralizing antibodies against the virus. One potential strategy is to combine SVA with cytotoxic peptide prodrugs. Because SVA’s 3Cpro already has protease activity, it bypasses the need for the addition of additional enzymes into the cell. Once the virus enters the tumor cell, 3Cpro could cleave the peptide prodrug to produce toxic peptides, leading to targeted tumor cell death that can also influence cytotoxicity to adjacent uninfected cells through the “bystander effect” (112). On the other hand, the production of neutralizing antibodies can be delayed by reducing the immunogenicity of SVA. For example, a mutation in VP2 protein at the virus-receptor boundary (S177A) can significantly promote virus replication and modify the exposed residues on the surface of the capsid that are not involved in receptor recognition to suppress the immune response, thereby improving the efficacy of SVA and reducing its immunogenicity (7).

As previously mentioned, the immune responses of the body are usually a double-edged sword: on the one hand, they limit the repeated administration of OV, while on the other, they can promote the body’s anti-tumor properties. How to enhance the anti-tumor aspect of OV and weaken the body’s neutralization of the virus is an important breakthrough point. There are a variety of OV designs that can perform multiple immunomodulation, such as encoding TAAs in OV vectors to create therapeutic drugs with the characteristics of OV and cancer vaccines (113). Taking into account the heterogeneity of tumor cells, viruses using multiple TAAs combinations can broaden the target spectrum of activating anti-tumor T cells. OVs can also be designed to mediate direct contact between T cells and tumor cells as bispecific T cell junctions (BiTEs) or membrane integrated T cell junctions (MiTe), and their expression in infected cells can lead to local T cell activation and tumor cell killing (28, 72). At the same time, OV activates the body’s immunity to turn the “cold” tumor microenvironment into “hot”, turning them into a microenvironment suitable for anti-tumor immune activation. When combined with ICIs or CAR-T, it can achieve complementary effects and maximize the anti-tumor efficacy (28, 114). Although multiple uses of the same OV will make the body produce neutralizing antibodies against the virus, the use of two different OVs successively can focus the patient’s immune response on TAAs and stimulate the body in the form of “ Prime-Boost” to produce stronger anti-tumor immunity. This multi-virus combination therapy may circumvent or reduce the antiviral immune response and prolong OV transmission in tumors (95, 115). At present, nanobody-cytokine fusion molecules have been proven to exert stronger anti-tumor activity through a variety of mechanisms to kill tumor cells (116). This multifunctional fusion molecule is also promising to be used in OVs.

## Concluding Remarks

Previous studies have shown the potential of SVA as an oncolytic virus, and thus its use for the treatment of cancers is encouraging. Although SVA is a natural OV, genetic manipulation is needed to satisfy the regulatory safety and efficacy needs. In addition, the combination of SVA with other treatment methods could greatly enhance the efficacy of OV, which can beneficially regulate the tumor immune landscape. To date, the availability of neutralizing antibodies produced at large scale remains an important challenge affecting its development in the clinic. In view of this situation, future research could benefit on improving the immune activation characteristics and delivery system of OV to achieve better therapeutic effects. An in-depth understanding of the exact mechanisms at the interaction between SVA and the host immune system will aid in the discovery of better strategies for anti-tumor immunity. Moreover, the important receptor TEM8 can be used to determine more tumor types that SVA can target and expand its breadth of potential uses. Further research on the reasonable combination of SVA and other therapies is expected to find the combination strategy to achieve the optimal therapeutic effect and minimal therapeutic toxicity for patients.

## Author Contributions

Conceptualization: DL and HW. Writing-review and editing: DL, HW, QW, WL, BL, and DX. Supervision: YY and BM. Funding acquisition: BM. All authors have read and agreed to the published version of the manuscript.

## Funding

This research was funded by the National Natural Science Foundation of China (grant no.81602337) and Education Department of Heilongjiang Province (grant no.UNPYSCT-2017062), and the Natural Science Foundation of Heilongjiang Province (grant no.LH2021H050).

## Conflict of Interest

The authors declare that the research was conducted in the absence of any commercial or financial relationships that could be construed as a potential conflict of interest.

## Publisher’s Note

All claims expressed in this article are solely those of the authors and do not necessarily represent those of their affiliated organizations, or those of the publisher, the editors and the reviewers. Any product that may be evaluated in this article, or claim that may be made by its manufacturer, is not guaranteed or endorsed by the publisher.
